# Kinetic and Thermodynamic Study of Methylene Blue Adsorption on TiO_2_ and ZnO Thin Films

**DOI:** 10.3390/ma16124434

**Published:** 2023-06-16

**Authors:** William Vallejo, Carlos Enrique Diaz-Uribe, Freider Duran

**Affiliations:** Grupo de Fotoquímica y Fotobiología, Universidad del Atlántico, Puerto Colombia 81007, Colombia; carlosdiaz@mail.uniatlantico.edu.co (C.E.D.-U.); fgduran@mail.uniatlantico.edu.co.edu.co (F.D.)

**Keywords:** environmental remediation, thermodynamics, adsorption, thin films, TiO_2_, ZnO

## Abstract

In this work, we fabricated and characterized ZnO and TiO_2_ thin films, determining their structural, optical, and morphological properties. Furthermore, we studied the thermodynamics and kinetics of methylene blue (MB) adsorption onto both semiconductors. Characterization techniques were used to verify thin film deposition. The semiconductor oxides reached different removal values, 6.5 mg/g (ZnO) and 10.5 mg/g (TiO_2_), after 50 min of contact. The pseudo-second-order model was suitable for fitting the adsorption data. ZnO had a greater rate constant (45.4 × 10^−3^) than that of TiO_2_ (16.8 × 10^−3^). The removal of MB by adsorption onto both semiconductors was an endothermic and spontaneous process. Finally, the stability of the thin films showed that both semiconductors maintained their adsorption capacity after five consecutive removal tests.

## 1. Introduction

The world’s population growth and the energy and water requirements by industries (e.g., petrochemical [1], pharmaceutical [2], textile [3], agrochemical [4], fuels [5], plastics [6]) have caused a severe threat to the environment. Water pollution makes water unsafe for fauna and humans, affecting different environmental systems [7]. One of the challenges for this century is to ensure that the population has access to safe water; the Organization for Economic Co-operation and Development (OECD) recommends that governments encourage the joint management of water quantity and quality [8]. Various techniques for water remediation have been implemented in the last decades (e.g., physical, chemical, and biological treatment technologies) [9]. Among these methods, the adsorption method (a physical method) has received attention due to its low cost and its effectiveness in removing contaminants from water [10]. During the adsorption process, the pollutant is retained on the substrate surface. Adsorption can be described as a chemical (covalent bond) or physical (weak electrostatic interactions) interaction between an adsorbate and adsorbent surface [11]. Different materials have been used to apply the adsorption process (e.g., zeolites, [12], alumina [13], clay [14], active carbon [15], biomass [16], semiconductors [17], MOF [18]). In the literature, there are various reviews on dye removal by adsorption using different materials [19,20,21]. Metal oxides have two synergic properties: (i) they can act as an adsorbent and (ii) as antimicrobial agents [22]. Furthermore, because semiconductors have variable oxidation states, large surface areas (e.g., as nanomaterials), and great versatility, they can be used for environmental control and contaminant removal [23]. Khoshhesab et al. reported that nanoparticles of ZnO had 92.3% of adsorption capacity in the removal of Congo red from a solution (75 ppm) after 120 min of contact [24]. Syarif et al. reported that nanoparticles of CuO had 61.0% of adsorption capacity in the removal of methylene blue from a solution (5 ppm) after 10 min of contact with CuO nanoparticles [25]. Noreen et al. utilized Fe_3_O_4_ nanoparticles to remove a reactive blue dye from a solution, and reported 35 mg/g of adsorption capacity after 10 min of contact [26]. Abdullah et al. prepared MnO_2_ nanoparticles to remove methylene blue from an aqueous solution, and reported 22.2 mg/g of adsorption capacity after 60 min of contact [27]. ZnO and TiO_2_ are alternative adsorbents, as they are innocuous to the environment, they are chemically and physically stable, and have adequate surface properties (e.g., roughness, porosity, and surface area) [28,29].

Currently, in heterogeneous photocatalysis, as a previous step to the photocatalytic degradation process, the sorption/desorption equilibrium is required. However, the adsorption process studied is not commonly reported in photocatalytic studies [30]. Although there is a high potential of ZnO and TiO_2_ as adsorbents, there are few reports on the thermodynamic study of dye adsorption onto the surface of these semiconductors. In this contribution, we synthesized and characterized ZnO and TiO_2_ thin films and studied the kinetics and thermodynamics involved in the removal of MB by adsorption onto both thin films.

## 2. Materials and Methods

### 2.1. Synthesis and Characterization of Thin Film Deposition

We used ammonium hydroxide and zinc acetate in the synthesis of ZnO powders, according to the procedure described in a previous report [31]. We used Degussa powder (P25) (Sigma-Aldrich, 99.5%, St. Louis, MO, USA) as a source of TiO_2_ in the fabrication of TiO_2_ thin films, according to the procedure described in a previous report [32]. We immobilized all catalysts on solid substrate to solve problems regarding catalyst removal after finishing the photocatalytic procedure [33]. We utilized the Doctor Blade technique for thin film deposition: First, we prepared a mixture of ZnO or TiO_2_ powders, polyethylene glycol (PEG 5000) (Sigma-Aldrich, 99%, St. Louis, MO, USA), isopropyl alcohol (Sigma-Aldrich, 99%, St. Louis, MO, USA), and water. After suspension stabilization, the slurry was loaded into a soda lime substrate by the Doctor Blade method. Finally, the thin films were sintered at 500 °C for 1 h [31,34]. The thin films were characterized by diffuse reflectance spectroscopy measurements, providing information about the optical band gap energy of the semiconductors; by Raman spectroscopy assays, which allowed verifying the presence of ZnO and TiO_2_ in the coatings; by X-ray diffraction measurements, which provided information about the crystalline structure of the thin films; and by scanning electron microscopy (SEM) assays, which allowed verifying their morphological properties.

### 2.2. Adsorption Kinetic and Thermodynamic Study

The semiconductors’ films were immersed in a solution of methylene blue—MB (10 mL; 10 mg/L) (Sigma-Aldrich, ≥95%, St. Louis, MO, USA) contained in a glass batch reactor provided with an air bubbling system (0.5 L/min). The reactor was stored in the dark to study the MB adsorption process on the films. An aliquot was extracted at time zero and every 5 min thereafter for 50 min to determine the adsorption–desorption equilibrium time. We determined MB concentration by spectrophotometry at 665 nm using the Lambert–Beer law with a calibration curve (R^2^ = 0.997). We determined the adsorption capacity of MB on the semiconductors according to [35]:

(1) $$q_{t}=\frac{\left(\left(C_{0}-C_{t}\right)\cdot V\right)}{m}\;$$

where *q_t_* is the amount (mg) of MB adsorbed per gram of semiconductor (mg/g) at each time; *C*_0_ is the initial MB concentration (mg/L); and *m* (g) is the amount of semiconductor. We applied the pseudo-first-order (PFO) and pseudo-second-order (PSO) models to fit experimental data according to these equations [35]:

(2) $$ln\left(q_{t}-q_{e}\right)=ln\left(q_{e}\right)-k_{1}t$$

(3) $$\frac{t}{q_{t}}=\frac{1}{k_{2}q_{e}{}^{2}}+\frac{t}{q_{e}}$$

where *q_t_* is the amount of MB adsorbed per unit mass of the adsorbent (mg·g^−1^) at each time; *q_e_* is the maximum sorption capacity (mg ·g^−1^); and *k_1_* (min^−1^) and *k_2_* (g·mg^−1^·min^−1^) are the rate constants of the pseudo-first- and pseudo-second-order models, respectively. The fitting correlation coefficient (R^2^) was used to determine the best-fitting kinetic models. Finally, we calculated standard enthalpy (ΔH°), standard entropy (ΔS°), and standard Gibbs free energy (ΔG°) for the adsorption process applying the Arrhenius equation [36]:

(4) $$K=\frac{q_{e}}{C_{e}}$$

(5) $$\Delta G^{{}^{\circ}}=-RTlnK$$

(6) $$lnK=\frac{\Delta S^{{}^{\circ}}}{R}-\frac{\Delta H^{{}^{\circ}}}{RT}$$

## 3. Results

### 3.1. Raman Characterization

**Figure 1:**
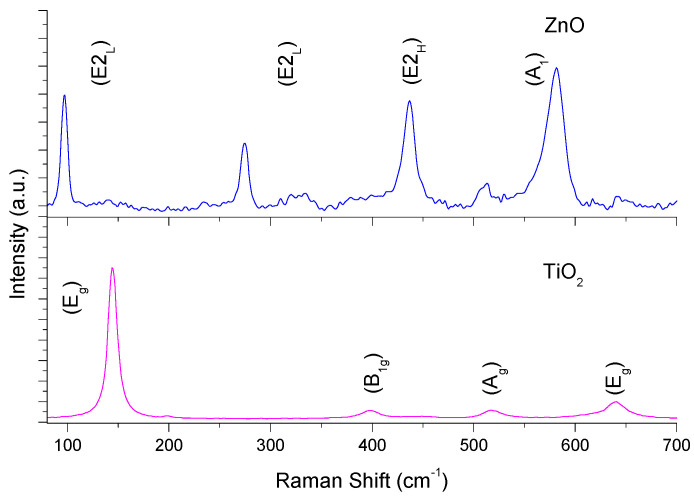
Raman spectrum of ZnO and TiO_2_ thin films.

The Raman spectroscopy results are shown in Figure 1. Six Raman-active vibrational modes were observed for TiO_2_ in Raman spectroscopy (e.g., A_1g_ + 2B_1g_ + 3E_g_) [37]. Three Raman-active vibrational modes were observed for ZnO in Raman spectroscopy (e.g., A_1_ + E_1_ + E_2_) [38]. Both catalysts shows the typical signals reported for such materials [39,40]. For the case of ZnO, the signals located at 274.5 cm^−1^ can be associated with oxygen vacancies into the semiconductor lattice [41,42]. 

### 3.2. Structural Characterization

**Figure 2:**
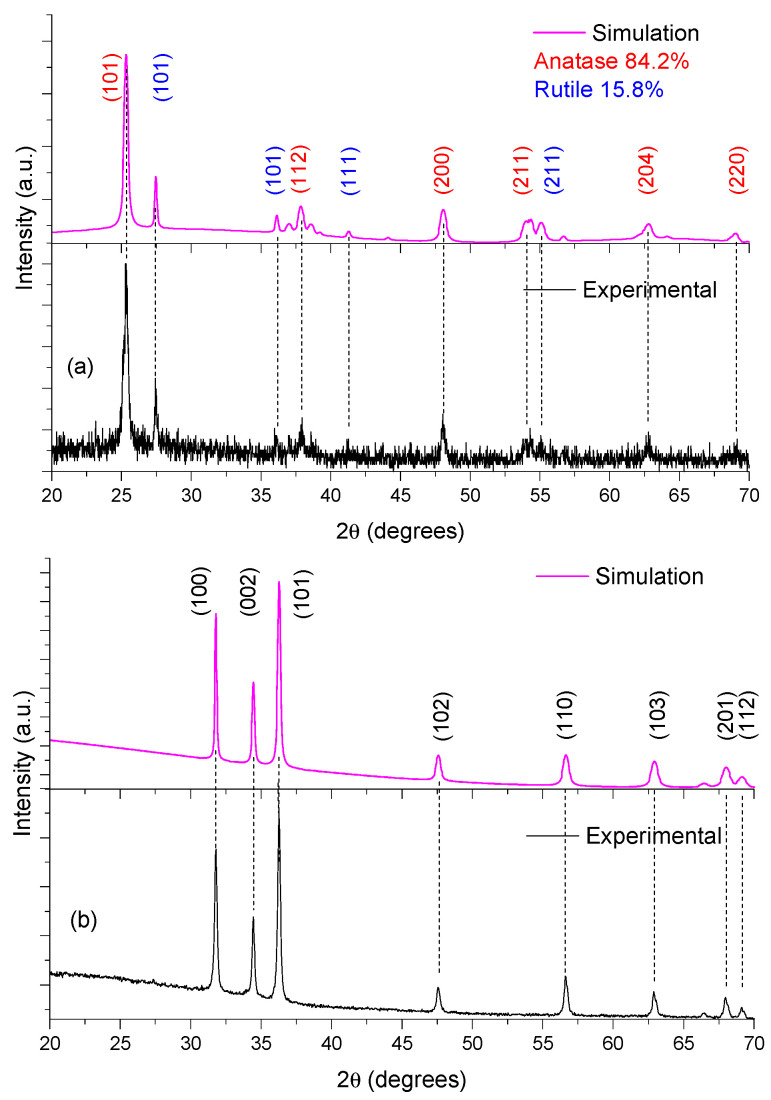
X-ray diffraction data and results of simulation for: (**a**) TiO_2_ and (**b**) ZnO.

Figure 2 shows the (experimental and simulated) structural results for both TiO_2_ and ZnO thin films. ZnO was polycrystalline, whose sample shows a plane of preferential growth located at 2θ = 36.27. This signal is assigned to plane (101), where ZnO thin films show six other preferential growth planes, with all these reflections corresponding to the hexagonal wurtzite phase (JCPDS No. 36−1451) [43]. For the XRD-TiO_2_ pattern, the TiO_2_ was polycrystalline and was formed by two different crystalline structures: rutile (JCPDS #021-1276) and anatase (JCPDS #071-1166). During thin film deposition, we utilized a TiO_2_ source (Degussa-P25), this material being a mixture of those two crystalline phases [44].

We utilized a PowderCell package to simulate the experimental XRD data. In the simulation, we employed the rutile and anatase forms of TiO_2_, and hexagonal wurtzite (ZnO) crystalline structures. We applied the Rietveld method (Bragg–Brentano geometry with the March–Dollase as model to preferred orientation; with the plane the plane (101) as plane’s orientation. The X-ray source was Cu $K_{\alpha }$ radiation (λ = 0.1544426 nm); the pseudo-Voigt 1 function iterations 300; and the φ factor was 1.9. This methodology was suitable to identify the crystalline phases in each thin film. Table 1 lists the crystalline parameters obtained from the simulations. We employed the Debye–Scherrer equation to determine grain size of the semiconductors [45]. The domain grain size of ZnO was 34.4 nm, and 24.1 nm and 38.8 nm for anatase and rutile structures, respectively. These results correspond to those of previous reports by other authors [44,46]. 

### 3.3. Morphological Characterization

**Figure 3:**
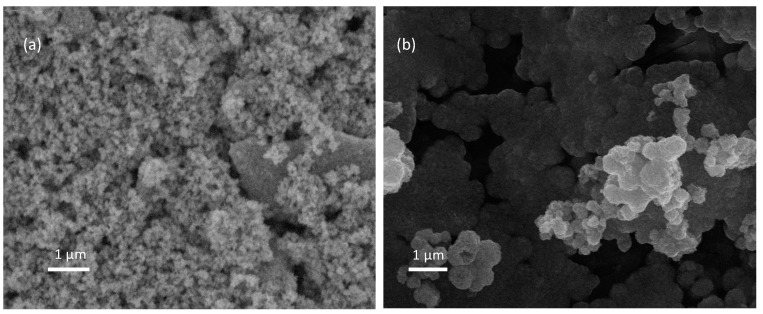
SEM images: (**a**) TiO_2_ thin films (×10,000); (**b**) ZnO thin films (×10,000).

Morphological properties are determined by the experimental conditions and deposition method [47]. We synthesized ZnO using the sol–gel method, and we utilized Degussa P25 as the TiO_2_ source. Figure 3 shows the morphological results for TiO_2_ and ZnO. These results show that the thin films’ surfaces are heterogeneous and porous, that TiO_2_ and ZnO are composed of microaggregates of different sizes, and that the agglomerated particles have two different spherical sized (50–80 nm to TiO_2_ and ~220nm to ZnO). Figure 3a shows typical morphological properties for Degussa P25 TiO_2_ [48]. The quasi-spherical ZnO nanoparticles are a commonly reported result when the sol–gel method is employed as a synthesis method [49]. Various authors have reported that the surface properties of the semiconductors are affected by synthesis method employed for their fabrication [23].

### 3.4. Spectroscopic Characterization

**Figure 4:**
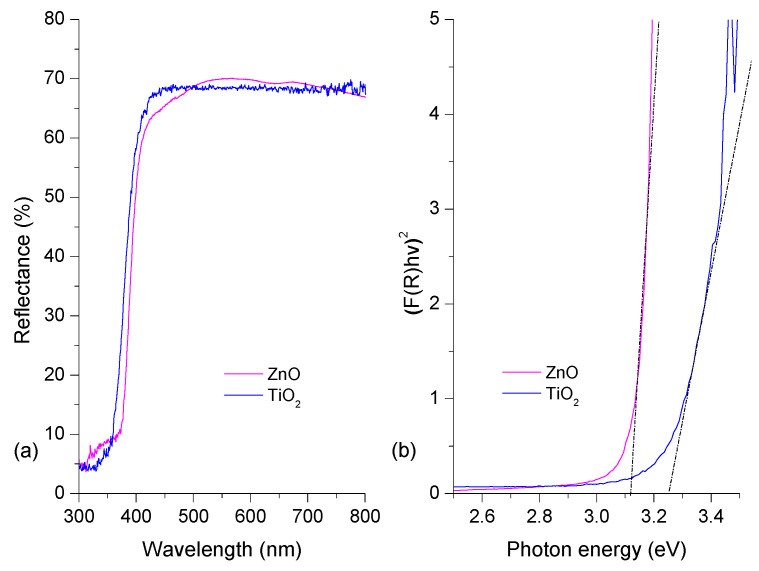
(**a**) TiO_2_ and ZnO thin films’ diffuse reflectance spectra; (**b**) Kubelka–Munk plots.

Figure 4 shows optical results for the ZnO and TiO_2_ semiconductors. Both of them show a high reflectance of approximately (or greater than) 60% after 360 nm. ZnO and TiO_2_ are not active under visible irradiation due to their high band gap (E_g_). We determined the E_g_ value using the Kubelka–Munk remission function [50]. Figure 4b shows the E_g_ estimation for each thin film. The estimated bad gaps for the thin films are shown in Figure 4b [51]. These results correspond to those of previous reports for ZnO and TiO_2_ Degussa P25 [52,53]. The spectroscopic and structural characterization verified the presence of ZnO and TiO_2_ in the coatings synthesized.

### 3.5. Adsorption Kinetic Study

The adsorption of dye onto a semiconductor surface is a principle that relies on two steps: (i) diffusion of reactants onto the semiconductor surface and (ii) adsorption of reactants onto the semiconductor surface. The first step (the diffusion process) (i) follows the classic laws of diffusion (e.g., Fick’s law) [54]. The second step (the adsorption process) (ii) can be a physical or a chemical process. During chemisorption, the dye molecule or ion attaches itself to a specific surface by a chemical bond and, in the physical adsorption, the dye molecules attach onto the adsorbent surface under the influence of van der Waals forces and hydrogen bonding [55]. The adsorption kinetic process can be studied through various theoretical methods (e.g., pseudo-first, pseudo-second, the intraparticle diffusion, Elovich) [35].

**Figure 5:**
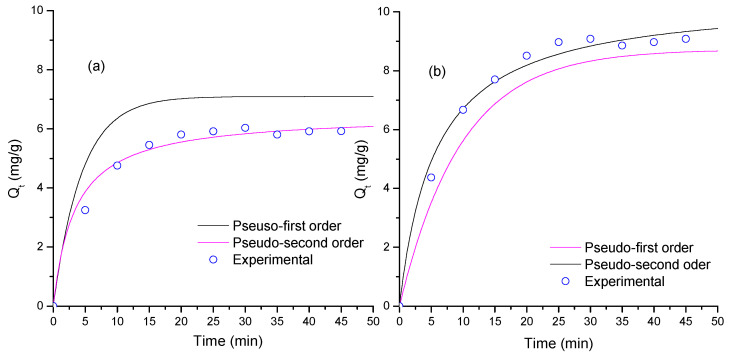
Adsorption kinetics and theoretical fitting of MB adsorption on thin films of the semiconductor oxides (**a**) ZnO and (**b**) TiO_2_.

Figure 5a,b shows the adsorption kinetics on TiO_2_ and ZnO. Figure 5 indicates that the TiO_2_ thin films reach 10.5 mg/g and the ZnO thin films reach 6.5 mg/g after 50 min of contact. These differences can be assigned to morphological properties and grain size. Table 2 lists the fitting results of the two models implemented. Table 2 indicates that the PSO model showed was suitable (greatest R value) to describe the adsorption process for both semiconductors. ZnO has a greater k_2_ value than that of TiO_2_ and a smaller *q_e_* value than that of TiO_2_, thus indicating that the ZnO surface saturates faster than the TiO_2_ surface, a behavior that can be associated to reduced grain size of TiO_2_ thin films. In the PSO model, the electrostatic interaction onto the surface affects the interaction with MB molecules. The MB dye is a cationic dye; the isoelectric point of TiO_2_ in water (7.0 [56]) is smaller than the isoelectric point of ZnO (9.5 [57]); and under experimental conditions, the ZnO surface is positively charged, then TiO_2_ would have more effective interaction with MB than ZnO thin films would. Furthermore, the grain size of TiO_2_ (anatase 84.2%) is smaller than that of ZnO (see Table 1), and the specific surface area of TiO_2_ should be greater than that of ZnO, increasing the MB adsorption capacity of TiO_2_ in comparison with that of ZnO thin films [58]. 

The adsorption capacities (AC) obtained for ZnO (6.49 mg/g) and TiO_2_ (10.5 mg/g) are suitable in comparison with previous reports. Dimauro et al. reported AC values of 7.0 mg/g, 7.4 mg/g, and 7.4 mg/g for MB adsorption onto V_2_O_5_, V_2_O_5_/SnO_2_, and V_2_O_5_/TiO_2_, respectively [59]. Debnath et al. reported an AC value of 9.6 mg/g for Congo red adsorption onto ZnO nanoparticles [60]. Singh et al. reported an AC value of 7.3 mg/g for MB adsorption onto Fe_3_O_4_ nanoparticles [61]. Song et. al. reported AC of 8.4 mg/g onto NiO nanoparticles [62]. Konicki et al. reported AC of BY28 and BR46 dyes onto Graphene Oxide was 68.5 and 76.9 mg/g, respectively [63]. Finally, the pseudo-second model has been reported by various authors as a suitable fitting model for dye adsorption on different adsorbent types. Table 3 lists reports fitting kinetic data with pseudo-second model. 

### 3.6. Adsorption Thermodynamic Study

**Figure 6:**
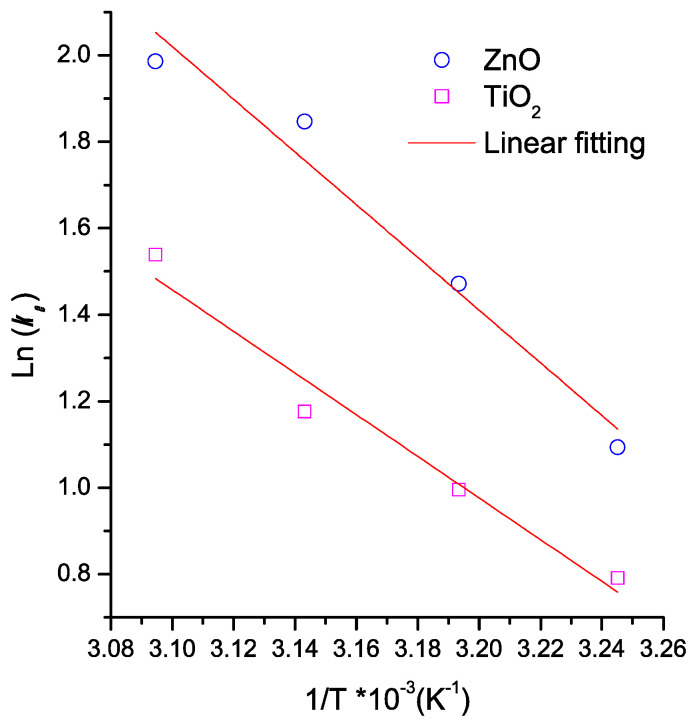
Thermodynamic calculation applying the Arrhenius equation to MB adsorption onto the semiconductor thin films.

Figure 6 shows the thermodynamic calculation applying the Arrhenius equation to MB adsorption onto the thin films of both semiconductors (Equation (6)). The ΔH° and ΔS° values were calculated from Figure 6. Table 3 lists the thermodynamic results. The removal of MB by using semiconductor oxides was a spontaneous process (ΔG < 0, for both materials). This result is due to the morphological properties of the semiconductors’ surface. Furthermore, the adsorption process was endothermic and more stable for TiO_2_ than for ZnO. The positive ΔS values of both semiconductor oxides could be associated with a degree of hydration of cationic MB molecules in the solution [64]. The MB remotion was more favored on TiO_2_ than on ZnO. Table 3 lists the thermodynamic results reported by other authors. Results show a variation range depending on both adsorbent and dye type. The ΔG° values for all studies listed in Table 3 are negative. It indicates that the dye adsorption onto adsorbents was spontaneous. This spontaneity of the process increases when the temperature increases. Bennabi et. al. reported that this behavior is associated with decreasing thickness of the boundary layer surrounding the adsorbent surface with temperature increasing. This effect improves the mass transfer of the dye to the adsorbent surface [65].

**Table 3:** Kinetic results for dye adsorption onto various materials.

Adsorbent/Dye	Temperature	Termodynamic Parameters		
		ΔG (kJ/mol)	ΔH (kJ/mol)	ΔS (J/mol)
* TiO_2 (this work)_	308 313 318 323	−2.90 −3.78 −4.65 −5.51	50.6	173
* ZnO _(this work)_	308 313 318 323	−7.12 −7.89 −8.65 −9.41	40.0	153
Graphene oxide/BY28 [63]	293 313 333	−1.69 −3.58 −5.47	2.74	16.5
NiO/Methyl orange [66]	303 318 333	−2.12 −2.41 −2.79	36.5	126
CuO/Methyl orange [66]	303 318 333	−1.65 −2.52 −3.38	15	58
Cu(I)−PANI/Orange16 [67]	303 308 313 318 323	−8.60 −8.77 −8.94 −9.11 −9.27	1.51	33.4
CdO/Congo Red [63]	298	−11.5	−−	−−
Chitosan/Congo Red [68]	298 308 318 328	−0.55 −2.45 −3.19 −2.41	34.5	118
Biochar/MB [69]	308 313 318 323	−0.95 −1.34 −1.74 −2.14	23.5	79.5
Actived carbon/MB [70]	298 308 318	−1.71 −1.91 −2.91	16.3	60.0
Biomass/MB [71]	298 308 318	−16.6 −19.6 −22.6	72.0	297

* Obtained from data of Figure 6.

Results verified that the adsorption process is an important step and indicated that such a process should be studied during photocatalytic tests. 

### 3.7. Recyclability Study

**Figure 7:**
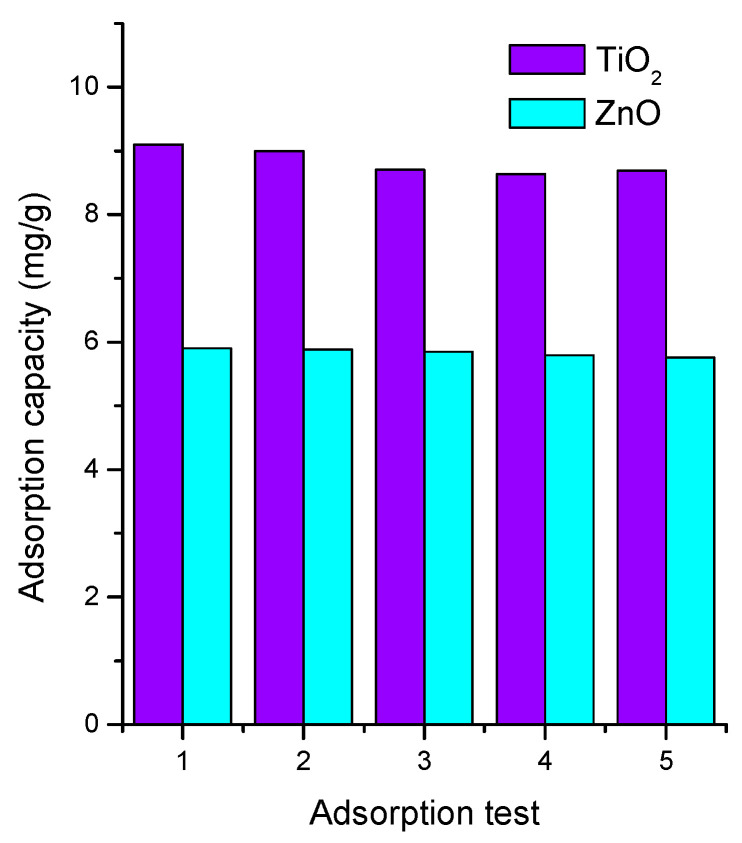
Stability test for MB adsorption onto TiO_2_ and ZnO.

To verify the potential application of semiconductors in continuous remediation water systems, we determined the recyclability of both semiconductor oxides in the MB adsorption during various cycles. Figure 7 shows the stability results of the studied semiconductors. The adsorption process was repeated five consecutive times. Figure 7 shows that after the fifth cycle, the removal performance reduced by 5% for TiO_2_ and 2% for ZnO. Such stable results are associated with the stability of the semiconductor oxides, and with the chemistry of the substrate (soda lime glass) and method of thin film deposition. These results indicate that the thin films were suitable and reusable for MB adsorption after five cycles.

These results are relevant to improve continuous flow remediation systems where adsorbents are incorporated in suspension form. Thin films can avoid additional separation steps, reducing the economic implementation of these systems.

## 4. Conclusions

We fabricated ZnO and TiO_2_ thin films. The morphological, optical, and spectroscopic characterizations verified the presence of ZnO and TiO_2_ in the coatings. Furthermore, the XRD simulation identified the crystalline structures of both semiconductors: TiO_2_ (anatase 84.2%—rutile 15.8%) and ZnO (wurtzite). The pseudo-second-order model was suitable to fit the kinetic results. Furthermore, TiO_2_ (*q_e_* 10.5 mg/g) was more effective in MB removal than ZnO (*q_e_* 6.5 mg/g). The MB adsorption onto both semiconductors was a spontaneous and endothermic process: TiO_2_ (ΔG = −2.9 kJ/mol; ΔH = 50.6 kJ/mol) and ZnO (ΔG = −7.1 kJ/mol; ΔH = 40.0 kJ/mol). Finally, the recycling test showed that the semiconductors were suitable after five consecutive adsorption tests. All the above results verified the significance of the adsorption process. The present authors consider that adsorption studies should be included during photocatalytic tests.

## Figures and Tables

**Table 1:** Structural properties of the sensitized semiconductor oxides.

Thin Film	Crystalline Plane	Grain Size (nm) ^1^	(a) ^2^	(c) ^2^
ZnO	(101)	34.4	3.2492	5.2044
TiO_2_—Anatase (84.2%)	(101)	18.5	3.7859	9.5044
TiO_2_—Rutile (15.8%)	(110)	65.3	4.5922	2.9568

^1^ Obtained from applied Debye–Scherrer equation to data of Figure 2. ^2^ Obtained from simulation PowderCell package.

**Table 2:** Kinetic results for MB adsorption on the semiconductor oxides sensitized.

Thin Film/ Model	1st Order *			2nd Order *		
	qe (mg g−1)	k1 (min−1) × 10−3	R2	qe (mg/g)	k2 (g mg−1min−1) × 10−3	R2
TiO_2_	6.89	112	0.885	10.5	16.8	0.993
ZnO	7.09	226	0.877	6.49	45.4	0.995

* Obtained from data in Figure 5.

## Data Availability

All relevant data are available within the article.
